# Spontaneous bladder rupture secondary to warfarin overdose: a case report

**DOI:** 10.1186/s12873-019-0294-6

**Published:** 2019-12-18

**Authors:** Taner Sahin, Ufuk Oner, Omer Baser, Ismail Kurtuncu

**Affiliations:** Deparment of Emergency Medicine, Kayseri City Hospital affiliated with University of Health Sciences, 38100 Kayseri, Turkey

**Keywords:** Warfarin overdose, Haematoma, Spontaneous bladder rupture, Surgery

## Abstract

**Background:**

Warfarin, a vitamin K antagonist, is a widely used medication for the treatment and prophylaxis of thromboembolic events. Patients with various clinical conditions due to warfarin overdose present to emergency departments. Although there may be serious bleeding due to a warfarin overdose, no bleeding may also be seen in some clinical conditions. Some of these bleedings may be life-threatening and result in death. Warfarin overdose and related cases of spontaneous bladder rupture are not frequently observed in the literature. We present a case of spontaneous bladder rupture due to warfarin overdose that was unexpectedly seen in a patient using warfarin for coronary artery disease and arrhythmia.

**Case presentation:**

A 77-year-old Caucasian male patient was admitted to the emergency department with abdominal pain, haematuria, and a reduced volume of urine lasting for three days. The patient’s amount of urine was reduced, and he came to the hospital for the first time with this complaint. The patient had local bruises on his arms and legs. From the ultrasound, retrograde cystography and computed tomography images, it was thought that there was blood accumulation due to bladder rupture to the intraperitoneal region. Spontaneous bladder rupture secondary to warfarin overdose was considered for this patient who also had an international normalized ratio (INR) level of 13.4. After the INR level was normalized with vitamin K and a prothrombin complex concentrate, the patient underwent surgery. During the operation, a catheter was placed in the bladder, and the bladder mucosa and muscle were closed separately with a primary repair performed by a urologist. The patient was discharged on the 8th postoperative day without any complications.

**Conclusion:**

In addition to the known findings of warfarin overdose in these patients presenting to the emergency department, we think that the emergency department staff should suspect bladder rupture, which is a fatal complication in the presence of signs such as oliguria, haematuria, anuria, abdominal pain, and syncope.

## Background

Warfarin, a vitamin K antagonist, is a widely used medication in the treatment and prophylaxis of thromboembolic events [1]. Patients with various clinical conditions due to warfarin overdose present to emergency departments [2]. In addition to the clinical condition of the patient, it is also important to revert the international normalized ratio (INR) level back within the normal range when treating warfarin overdose. Although there may be serious bleedings due to warfarin overdose, no bleeding may also be seen in some clinical conditions. Some of these bleedings may be life-threatening and result in death [3].

Bladder rupture may be observed if the structure of the bladder wall is damaged (due to radiotherapy, chronic cystitis, bladder cancer, etc.) or in cases of excessive bladder retention (neurogenic bladder, pregnancy, obstructive stone, etc.). Moreover, bladder rupture may also be seen because of trauma, an intravesical obstructive stone or a tumour or as a complication of prostate surgery, all of which can be in the patient’s recent history or occur spontaneously. After rupture, an acute abdominal presentation is seen [4, 5]. Spontaneous bladder rupture is most commonly observed after pelvic radiotherapy [6].

This study aimed to discuss a case in which a patient using warfarin for cardiac arrhythmias presented to the emergency department with haematuria, abdominal pain and a reduced volume of urine and was diagnosed with bladder wall rupture as a result of the examinations. As no other studies on bladder rupture secondary to warfarin overdose were found in the literature, we would like to emphasize the importance and specificity of our case study.

## Case presentation

**Fig. 1:**
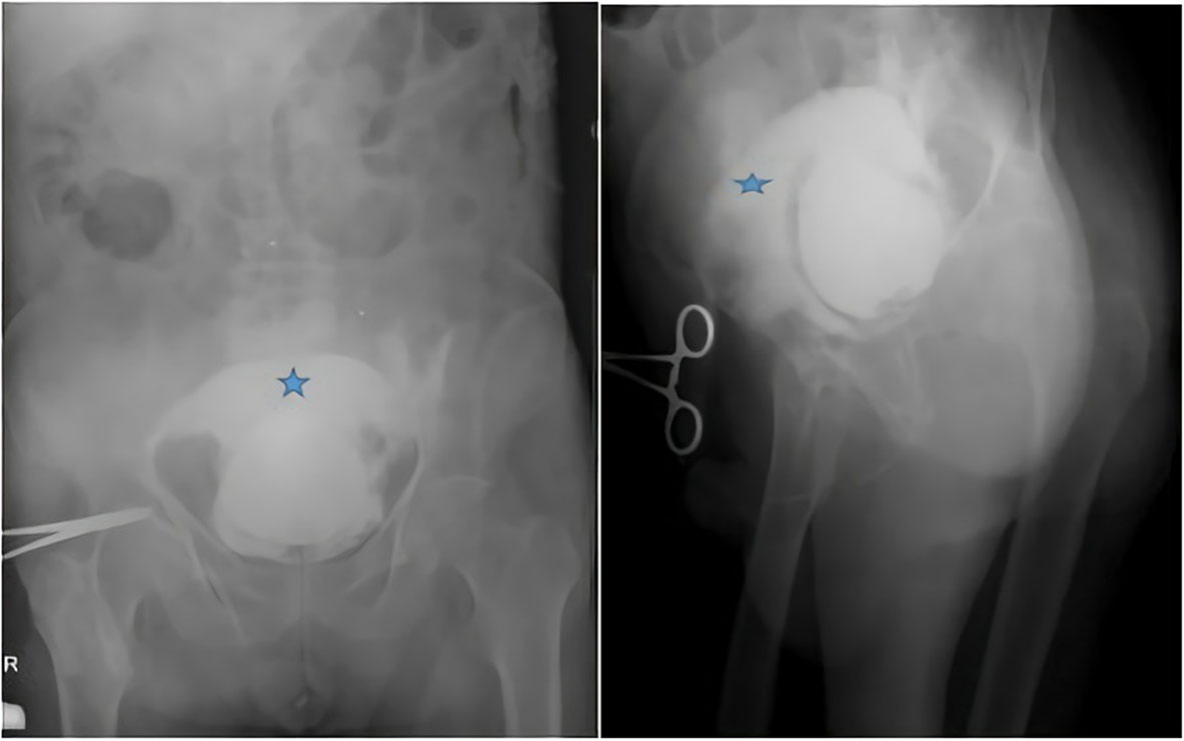
Direct AP and lateral cystography (*Contrast material leakage out of the bladder)

**Fig. 2:**
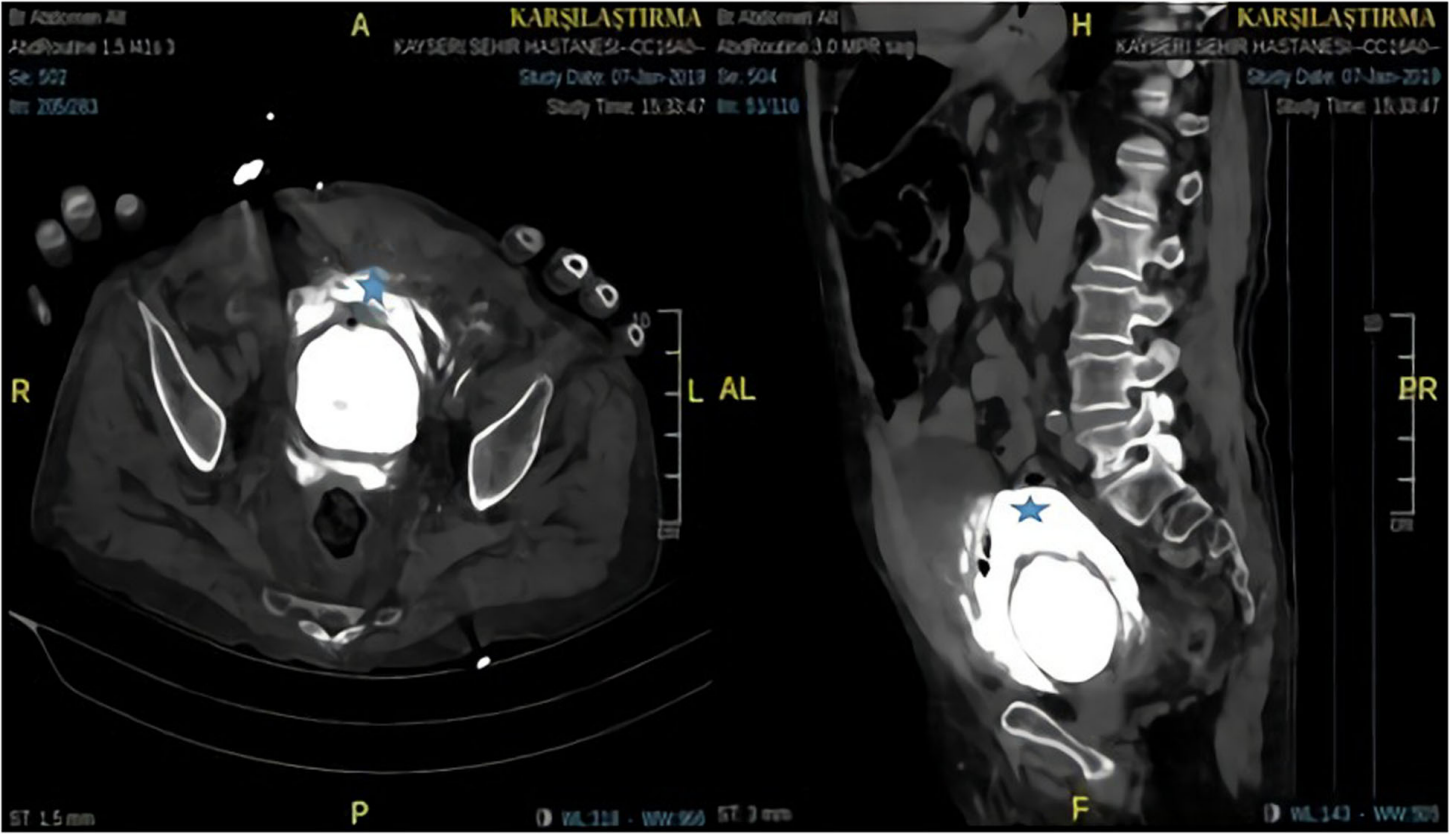
Abdominal CT with contrast images: axial and sagittal (*Contrast material leakage out of the bladder)

**Fig. 3:**
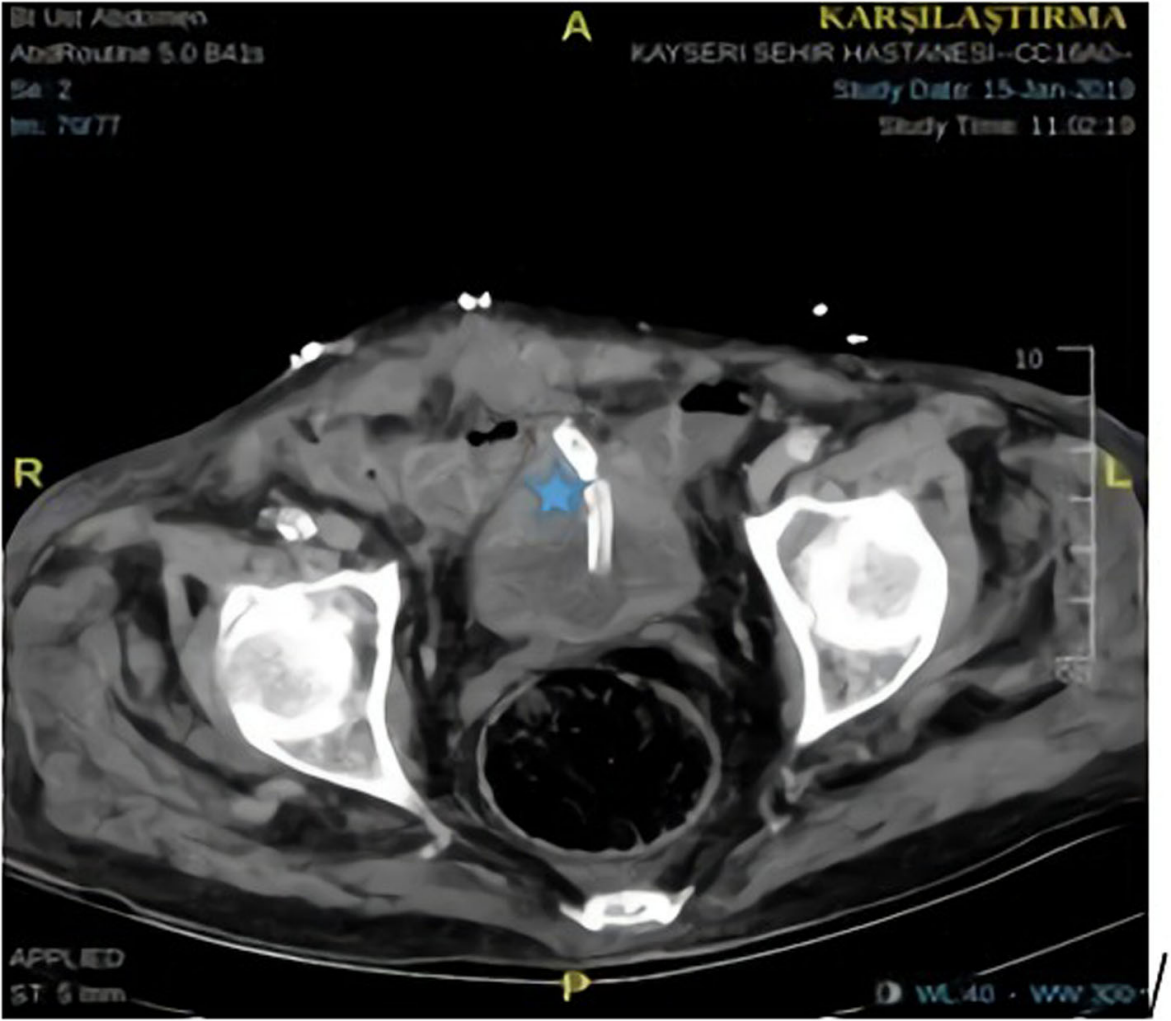
Pelvic CT axial image on the postoperative day 8 (*Foley catheter is seen in the bladder)

A 77-year-old Caucasian male patient was admitted to the emergency department by ambulance with abdominal pain, haematuria, and a reduced volume of urine lasting for three days. The patient’s history revealed that he normally urinated 5 or 6 times a day but that he had been able to urinate once a day for the last 3 days and that the amount of urine was reduced, he came to hospital for the first time with this complaint. The patient reported that he had used clarithromycin 500 mg tablet two times a day one week ago because of acute pneumonia. The patient used the following medications: inhaler (ß_2_- mimetic), Coraspin® 100 mg and warfarin for coronary artery disease (CAD), arrhythmia, hypertension and chronic obstructive pulmonary disease (COPD). He had no recent medical history of surgery, bladder cancer or trauma. Upon his physical examination, the Glasgow Coma Scale (GCS) was 15. The patient was oriented, cooperative and alert. His vital signs were as follows: blood pressure was 148/88, respiratory rate was 20, oxygen saturation obtained from the finger was 94% on room air, pulse was 92, and temperature was 36.6 °C. He had local bruises on his arms and legs. The abdominal findings were as follows: he had suprapubic tenderness to deep palpation in the bilateral lower quadrants. Double vascular access (wide lumen) was established, and a bladder Foley catheter was inserted. His urine output was reduced, there was a total of 50 ml of residual urine in the bladder, and gross haematuria was observed. We thought that the patient may have developed a urinary tract infection and acute renal failure as a result. Free fluid was detected in retrovesical space in FAST USG. To elucidate the ethology of acute renal failure, a urinary system ultrasound was requested, and retrograde cystography and abdominal computed tomography were performed to determine whether there was intraperitoneal or extraperitoneal injury to the bladder as well as fluid in the retrovesical area. Posterior-anterior chest X-ray and abdominal X-ray were requested for the patient. There were no major findings of free-air or perforation with direct radiography; therefore, cystography was performed with the use of a retrovesical opaque medium (Fig. 1). From the imaging, it was thought that there was blood accumulation due to bladder rupture to the intraperitoneal region. The laboratory results showed that his haemoglobin (Hb) was 13.9 g/dL, haematocrit (Ht) was 39.6%, platelet count (PLT) was 220 10^3/L, blood urine nitrogen (BUN) was 34.5 mg/dL, creatinine was 0.90 mg/dL, eGFR was 82 mL/min/1.7 m^2, prothrombin time (PT) was > 180 s, INR was > 12 and that the patient had gross haematuria. We thought that the use of clarithromycin in combination with warfarin heightened the effect of warfarin, resulting in an intra-bladder haemorrhage and subsequent risk for bladder rupture. These results were associated with warfarin overdose, and the patient was treated with a slow IV infusion of 10 mg vitamin K for 30 min. The patient was evaluated by urology and radiology specialists, and a haematoma was seen in his bladder with USG. Therefore, abdominal CT was performed to determine whether the rupture was intraperitoneal or extraperitoneal. According to the results of abdominal CT, spontaneous bladder rupture secondary to warfarin overdose was observed (Fig. 2). A slow IV infusion of 60 mL prothrombin complex concentrate (PPC) was administered to the patient over 45 min, and he was scheduled to undergo surgery with a preliminary diagnosis of bladder rupture after microscopic haematuria. The patient’s INR level was measured again after 1 h, and the result was 1.4. The patient was transferred for surgery. During the surgery, organized haematoma in the bladder and a perforation area of 2–3 cm in the posterior wall of the bladder were detected. A catheter was placed, and the bladder mucosa and muscle were closed separately with a primary repair performed by an urologist. After the bladder repair, there were no unanticipated events, and the patient was transferred to the intensive care unit. The urine output of the patient was 530 ml on the first postoperative day, 950 ml on the second postoperative day and 1600 ml on the third postoperative day. On the 8th day of his postoperative stay, an abdominal CT was performed again for quality control, and the results showed no complications (Fig. 3). The patient’s complaints improved. Therefore, the patient was transferred to the ward. The patient was hospitalized for a total of 8 days. During this period of follow-up, his anticoagulant level was adjusted, and the patient was discharged and made a full recovery.

## Discussion and conclusion

Spontaneous bladder rupture is most commonly observed after radiotherapy to the pelvic area [5]. It is generally observed in patients who have recently undergone transurethral resection [4, 5]. Bladder rupture may also be seen in cases in which the structure of the bladder wall is damaged (due to radiotherapy, chronic cystitis, bladder cancer, etc.) or in cases of excessive bladder retention (neurogenic bladder, pregnancy, obstructive stone, etc.) [4]. Patients can present to the hospital with gastrointestinal bleeds, haematuria, intraoral bleeding, nosebleeds or subcutaneous bleeding as a result of warfarin overdose. While some of these events may be minor bleeding events that cause simple bruising, some may also be life-threatening major bleeding events. To treat warfarin overdose, vitamin K, fresh frozen plasma (FFP) and PCC can be used [3, 6].

Spontaneous bladder rupture with acute abdomen requires emergency surgical intervention [4]. The American Urological Association (AUA) guidelines recommend that intraperitoneal bladder ruptures be surgically repaired. Unrecognized and unrepaired intraperitoneal bladder ruptures may lead to peritonitis, sepsis, and renal failure. The AUA guidelines recommend that uncomplicated extra-peritoneal bladder injuries should be managed conservatively with catheter placement. Standard therapy involves leaving the catheter in place for 2 to 3 weeks, but it may be left longer in some cases. Extra-peritoneal ruptures that do not heal after 4 weeks of catheter drainage should be considered for surgical repair [5].

Patient admissions to emergency departments due to haematuria and minor or major bleedings secondary to warfarin overdose are very common. In the treatment of these patients, an early diagnosis of major bleeding, which is life-threatening, and immediate intervention, as well as clinical stability of the patient are the top priorities. Patients with warfarin overdose are mostly discharged from the emergency department after reaching an optimal INR level by receiving vitamin K, FFP or PCC. However, the conditions of these patients after leaving the emergency department are not known by emergency physicians. This is why patients should be recommended to re-visit the emergency department soon for INR level measurements or evaluations of possible bleeding in any part of the body during discharge [7].

In our case, warfarin combined with aspirin may have led to a predisposition to bleeding into the bladder. We could not prove this exactly, but we considered that spontaneous rupture occurred due to haematoma caused by warfarin use since the patient had not recently undergone surgery on his prostate or bladder, he had no history of radiotherapy to the pelvic area, and he was not diagnosed with BPH (benign prostate hyperplasia). Early and appropriate diagnosis and immediate surgical intervention prevented the possible mortality of this patient in the emergency department. After a stay in the intensive care unit, follow-up visits in the ward and adjustments of the warfarin dose, the patient was discharged from the hospital and made a full recovery.

The use of alcohol, some antibiotics (penicillin, cephalosporin, chloramphenicol, trimethoprim-sulfamethoxazole, ciprofloxacin, erythromycin, clarithromycin, sulphonamides, etc.), pain medications, various drugs and foods may increase or decrease the effect of warfarin [8]. In our patient, we think that the warfarin effect was increased as a result of using warfarin with clarithromycin and caused subcutaneous bleeding in the bladder.

We have not encountered any studies on spontaneous bladder rupture secondary to warfarin overdose in the literature, which emphasizes the importance of our case. In patients with a delayed diagnosis and treatment due to acute abdomen secondary to bladder rupture, the condition can be highly life-threatening. In addition to the known findings of patients who present to the emergency department with warfarin overdose, we think that the emergency department staff should suspect bladder rupture, which is a fatal complication in the presence of signs such as oliguria, haematuria, anuria, abdominal pain, and syncope.

## Data Availability

Data sharing is not applicable to this article as no data sets were generated or analysed during the current study.

## Abbreviations

AUA: American Urological Association
BPH: Benign prostate hyperplasia
CAD: Coronary artery disease
COPD: Chronic obstructive pulmonary disease
CT: Computerized tomography
FAST: Focused Assessment with Sonography for Trauma
FFP: Fresh frozen plasma
GSC: Glasgow Coma Scale
INR: International normalized ratio
PCC: Prothrombin complex concentrate
USG: Ultrasonography
